# Space-Time Surveillance of Negative Emotions after Consecutive Terrorist Attacks in London

**DOI:** 10.3390/ijerph17114000

**Published:** 2020-06-04

**Authors:** Dajun Dai, Ruixue Wang

**Affiliations:** 1 Department of Geosciences, Georgia State University, Atlanta, GA 30303, USA; rwang34@ncsu.edu; 2 Center for Geospatial Analytics, North Carolina State University, Raleigh, NC 27695, USA

**Keywords:** terrorist attack, disaster, Twitter, social media, GIS

## Abstract

Terrorist attacks pose significant threats to mental health. There is dearth information about the impact of consecutive terrorist attacks on space-time concentrations of emotional reactions. This study collected (1) Twitter data following the two terrorist attacks in London in March and June of 2017, respectively, and (2) deprivation data at small areal levels in the United Kingdom. The space-time permutation model was used to detect the significant clusters of negative emotions, including fear, sadness, and anger in tweets. Logistic regression models were used to examine the social deprivation of communities associated with negative tweeting. The results reported two significant clusters after the March attack, one was in London, ten days after the attack, and the other was far from the attack site between Manchester and Birmingham, three days after the attack. Attention to the reoccurring attack in June diminished quickly. The socially deprived communities experienced double disadvantage—sending fewer tweets but expressing more negative emotions than their counterparts. The findings suggest that terrorism can affect public emotions far and broad. There is a potential for surveillance to rapidly identify geographically concentrated emotions after consecutive or prolonged disasters using social media data.

## 1. Introduction

Terrorism continues on all scales [1,2] and has substantial consequences on mental health in public [3,4,5]. Research to date has documented a variety of short- or long-term mental health effects in the aftermath of disasters [6,7]. The impact could range from mild sadness, depression, fright, and post-traumatic stress to long-term post-traumatic stress disorder (PTSD), among others [7,8,9]. Traditionally, researchers rely on surveys and interviews [6,10] to understand people’s reactions to disasters. In recent years, social media data have seen their increased usage in disaster research, such as terrorist attacks [3,11,12], earthquakes [13,14], and hurricanes [15,16]. As a rich crowdsource of data, these social media platforms have contributed substantially to the understanding of news, thoughts, and ideas among social media users after disasters [11,16]. Social media messages- thus provide great potential to understand emotions during and after terrorist attacks.

Terrorism is intended to gain a political or social objective by provoking fear of a targeted audience through violence [17,18,19]. Thus, mental suffering is usually more prevalent than physical injuries [20], not only to these who directly experience the event but also to others who are exposed through news and images. It has been a global issue with more than 190,000 events since 1970 [21]. The threat has been growing, especially after the September 11 attack in the United States [22]. Besides destructions and physical harm, previous research has documented a wide range of mental consequences by terrorism, including low birth weight [23], traffic fatalities [24], panic disorder, helplessness, fear, horror, anxiety, depression, PTSD [6,7,9,20], among others. The majority of the people may experience mild distress such as insomnia or sadness and may benefit from education and community-wide supportive interventions [7,20]. A small group may develop more moderate symptoms or major depression [20]. Compared to natural disasters, mass violence events such as terrorism were significantly more likely to result in more persistent psychopathology [25], more severe impairment, [26,27,28], and higher rates of diagnosable mental illnesses [27,28].

Although many studies provide an in-depth understanding of the mental health resulting from terrorism, there is dearth information about the dynamics of geographic concentrations in mental suffering in the event of consecutive attacks. The epidemiological studies above [6,7,20] have discerned a spectrum of aberrant mental reactions after terrorist attacks. However, these studies provided little information about where and when stress reactions are concentrated. Previous studies used geotagged social media data coupled with geospatial technologies in the Geographic Information Systems (GIS)—to study terrorist attacks, yet research is still needed. For example, Cvetojevic and Hochmair [11] analyzed the propagation of geo-located tweets that were posted in response to the deadly 2015 Paris terrorist attacks. However, they did not reveal the health consequences of the disaster. Analyzing geotagged tweets in Paris in the first three days after the same attack, Gruebner and others [3] identified significant clusters of fear around three attack sites two days after the disaster. The research was applauded in presenting the potential of GIS in mental health research. However, there were vagaries of emotional variations in a broader region beyond three days. Furthermore, the characteristics of the communities were unclear where the negative emotions reside. In fact, as most disaster research rely on a single traumatic event, understanding people’s reactions in space and time to consecutive terrorist attacks remains elusive. 

Inspired by the current research need in disaster research and the increasing terrorist threats, this study has two objectives. First, it identifies the areas of particularly pronounced emotional reactions after two terrorist attacks in London in 2017 using geotagged tweets. Second, it investigates the social characteristics of neighborhoods where tweets presenting negative emotions were sent. Addressing the first question will help to assess how the public responded among Twitter users and locate communities in need after disasters. Identifying these communities will enable the assessment of time-specific changes in the intensity and duration of distress locally [12]. This not only assists policymakers and health professionals in allocating resources to the places where they are needed most but also contributes to the pressing need to highlight the affected areas that require additional study [3,7]. Addressing the second question will bring a better understanding of how terrorism disproportionally affects socially deprived groups in a society.

## 2. Study Area, Data, and Methods

### 2.1. Study Area

The study area was the United Kingdom, which consists of England, Wales, Scotland, and Northern Ireland. The UK has been a frequent target of terrorism in Europe in recent years. In 2017, there were four violent attacks, and three occurred in London (1) Westminster attack on 22 March when an Islamist drove a car into pedestrians on Westminster Bridge, killing four and injuring more than 40, (2) London Bridge attack on 3 June when three Islamists drove a van into pedestrians on London bridge, before stabbing people causing eight deaths and nearly 50 injuries, (3) Finsbury Park attack on 19 June when a British man drove a van into Muslim worshippers killing one and injuring at least nine people, and (4) Parsons Green bombing when a bomb exploded on a train on 15 September injuring 30 people. This study focused on the first two attacks because they were similar in the nature of attacks in a short period—both used vehicles to randomly attack pedestrians in close proximity, making the assessment of public emotional reactions comparable.

### 2.2. Tweet Collection

We based our analysis on Twitter data. A tweet consists of 280 characters. With the increasing use of location-based techniques and smart devices, geotagged tweets become a major social media data source to understand mental health [12,15,16]. Although Twitter users are not representative of the entire population, Coppersmith and others [29] argue that these social media data provide a diverse set of signals related to mental health observable in tweets.

We created a Twitter account to collect tweets and to manage Twitter applications on the Twitter Apps platform (https://developer.twitter.com/). Within each Twitter application, a consumer key and an access token were assigned to users for retrieving tweets with Twitter Application Programming Interface (API). Both the consumer key, i.e., API key, and the access token, were required for each independent request. This study used the Tweepy API (https://www.tweepy.org), a comprehensive Python (Python Software Foundation, Wilmington, DE, USA) library for accessing the entire Twitter REST API method. A Python programming script was developed to extract tweets between two periods (3/22/2017–4/12/2017 and 6/5/2017–6/21/2017). For the March attack, we used two keywords, i.e., “London attack” and “UK Parliament attack”. Each keyword was used to make an independent request. For the June attack, we used the “London attack” and “London Bridge attack” as the keywords to retrieve relevant tweets. The scripts returned all tweets that contained the specific keywords regardless of their upper/lower case, word order, and the locations. Consequently, there were 165,881 tweets in March and 71,154 in June worldwide, from which we extracted geotagged tweets in the UK. Each tweet had a date, time, and content. In addition, if a tweet was geotagged, latitude/longitude was attached with each message. Tweets were considered duplicate if tweets carried the same message from the same user at the same time and location, and thus only one tweet was retained. However, if two identical tweets were from the same location but at different times, both were kept because of the unknown purposes behind the Twitter user sending repeated messages. 

To examine the space-time variation in public emotional reactions to these two attacks and to identify the clusters of negative emotions, this study used the geotagged tweets in the UK as the study objects. The contents of the tweets and the geotag location associated with each tweet in the UK were retrieved. Using the time 0:00 on 22 March as the initial timestamp, we calculated the number of hours when a tweet was posted since this starting point in the timescale. For example, two tweets at 16:32 on 22 March and 20:54 on 5 June received 17 and 1,821 under the relative time, respectively.

One issue was that this tweet dataset might be incomplete because of the rate limit that Twitter sets. This rate limit restricts how many times the Twitter API can be used per user access token, which allows for 15 requests per window per access token, either 15 calls every 15 minutes or 180 calls every 15 min [30]. The API will return a “Too Many Request” response code when the script exceeds the rate limit. In the first day or two after each attack, high volumes of tweets were generated, and some tweets might have been missed between requests. Another challenge was that the use of keywords possibly excluded related tweets. The attempted searches using the bounding-box method without keywords were unsuccessful given limited computer processing power and memory. 

### 2.3. Analysis of Negative Emotions in Tweets

We dichotomized tweets based on the emotions in the content to determine twitter users’ attitudes towards the attacks. Previous studies identified negative emotions as fear, sadness, anger, shock, confusion, disgust, shame, among others [3,31]. Tweets were coded as 1 (negative) if they had any of these opinions related to the attacks. The rest of the tweets received 0 if they did not express these negative emotions, such as a tweet of the related news. This process is commonly accomplished through sentiment analysis using text-mining techniques, such as supervised or unsupervised machine learning algorithms [32,33,34,35,36,37]. However, these algorithms typically require a large sample dataset to develop classification rules, use the rules to classify the remaining tweets, and then evaluate the performance of these classifiers. Given the small size of the geotagged dataset in this research, we manually slogged through all tweets to assess their negative emotions. To minimize personal bias, we crosschecked all geotagged tweets among three researchers. Table 1 shows a sample of the geotagged tweets.

### 2.4. Cluster Detection of Negative Emotions

In order to understand the dynamics of negative emotions over space and time, we employed the space-time permutation model in SatScan (Kulldorff and Information Management Services Inc., Rockville, MD, USA-) [38]. We assumed that negative emotions are elevated after disasters with the loss of lives or property damage, especially caused by intentional harm [9,10]. Understanding the dynamics of the negative emotional consequences of terrorist attacks after combing through social media data would identify populations at risk early. This approach would help to detect geographically concentrated emotional reactions at a much lower cost compared to lengthy conventional surveys.

The space-time permutation model in SatScan was designed to analyze space-time data in order to identify disease clusters and, if detected, to determine the statistical significance of the clusters. SatScan provides three models for count data—the Poisson model, Bernoulli model, and space-time permutation model. In line with previous studies [3,39], we used the space-time permutation model because it requires case data only, i.e., the location and time of each negative tweet with no information needed about controls or a background population at risk. Under the null hypothesis, the negative tweets were independently distributed in a geographic area and during a specific time period. The alternative hypothesis was that a higher proportion of negative tweets were clustered at some geographical areas in a specific hour(s). The number of negative tweets in a cluster is compared to what would have been expected if the time and location of all negative tweets were independent of each other using the likelihood ratio test (Equation (1)).

(1) $${\left(\frac{c}{E\left[c\right]}\right)}^{c}{\left(\frac{C-c}{C-E\left[c\right]}\right)}^{C-c}$$

where *C* is the total number of negative tweets, *c* is the observed number of negative tweets within an area at a time period, and *E[c]* is the expected number of negative tweets within the area at the time period under the null hypothesis. *C−E[c]* is the number of negative tweets expected outside the area and the time period. When there is no covariate, the expected number of negative tweets at each area/time period is proportional to the number of tweets within this area and time period.

To search clusters, Satscan imposes a moving cylinder in the study area and time. The cylinder becomes an interval in a pure time search or an ellipse in a pure spatial search. This cylindrical search window moves across the study area and gradually changes in size and height to account for the number of cases inside the window at each location. It scans the area for an unusual increase in negative tweets of any area and size at the potential one-hour intervals, as well as multiple hours. The program maximizes the likelihood of all windows, and the one with the maximum likelihood is the most likely cluster. Under the null hypothesis, Satscan repeats the same analysis 999 times using random replications of the data. It then uses Monte Carlo hypothesis testing [40] to obtain the *p*-value of this most likely cluster by comparing the rank of the maximum likelihood from the real tweet data with the maximum likelihoods from the random replications. Assuming the rank from the real tweet data is *R*, the *p*-value will be *R/(1+999)*. Therefore, if the rank is within the top 50 of all maximum likelihoods from the random replications, the *P*-value of this most likely cluster in the real tweet data will be statistically significant (*p* < 0.05). A detailed explanation of the computation is available elsewhere [38]. The maximum search window size was selected as 75 km. To examine how sensitive the result was, this study alternated the search window size to 36 km and 100 km.

### 2.5. Social Characteristics Associated with Negative Tweeting

Multiple contextual variables were used to understand the social characteristics associated with negative tweeting in the four countries, i.e., England, Wales, Scotland, and Northern Ireland. We analyzed the social characteristics based on an index of multiple deprivations constructed in the four countries in 2015 [41] at the unit level of Lower-layer Super Output Areas (LSOA), respectively. LSOAs are geographic hierarchy to improve the reporting of small-area statistics in the UK [42]. Each LSOA, consisting of four to six output areas (OAs), has a population of 1,500 on average [42], similar to the population size of a census block group in the United States [43]. Conceptually output areas in the UK are equivalent to the US census blocks. The statistics provide a fine understanding of the social characteristics of the communities. The index amalgamates data from employment, income, education, skills and training, health and disability, crime risk (i.e., personal and material victimization, such as homicide and burglary), barriers to housing and services, and living environment in each LSOA. Each country ranks all LSOAs in its jurisdiction, with a rank of 1 being the most deprived. Rather than using the overall index of multiple deprivations, we utilized the rank orders in the original indicators in four domains (employment, income, education, and crime) among all domains based on three considerations. First, the other domains are fraught with problems of inconsistent measurements adopted by the four countries, and thus were excluded. Second, rather than a single index mingling various domains, the raw indicators allow us to discern the specific four aspects of social characteristics in an LSOA. Third, rank orders capture the position of an LSOA in the entire United Kingdom as opposed to raw measures. For example, the purchasing power of the same income is much higher in a rural village than that in London.

We scaled the rank order of an LSOA to a number between 0 and 1 in order for all ranks across the four countries to be comparable, given that the four countries have different numbers of LSOAs. Take the income deprivation as an example, for LSOA *i* in country *j*, its rescaled income deprivation rank order *D_ij_* is based on the Equation (2):

(2) $$D_{ij}=\frac{R_{ij}-1}{R_{max,j}-1}$$

where *D_ij_* is the rescaled income deprivation rank order, *R_ij_* is the original rank order of LSOA *i* in the income domain, *R_max,j_* is the highest rank order, that is, the total number of LSOAs in country *j*. After the rescaling, *D_ij_* will be a value between 0 and 1, where zero means the most deprived LSOA in terms of income in the entire UK.

The LSOAs were further categorized into four quartile groups based on their scaled rank orders. The four quartile groups reflect the most deprived, moderate deprivation, moderate affluence, and the most affluent LSOAs. The lowest quartile represents the most deprived group of LSOAs in one aspect of the four social characteristics, and vice versa.

To examine the association between the negative tweets and the four main categories of social characteristics, i.e., income, employment, education, and crime, this research used the logistic regression model in keeping with previous studies on social media analysis [44,45]. The dependent variable used binary coding to represent being negative (code 1, negative) for a tweet’s emotions (see Equation (3)). This study, therefore, models the likelihood of presenting negative emotions in a tweet. To compare the change of the likelihood after consecutive attacks, models 1 and 2 considered tweets related to March and June attacks, separately.

(3) $$log\frac{p}{1-p}=\beta_{0}+\overset{4}{\underset{i=1}{\sum }}\beta_{i}\cdot x_{i}+\varepsilon$$

where *p* is the probability for a tweet to present negative emotions, *x_i_* represents one of the four rescaled deprivation rank orders as an independent variable, *β_i_* represents the coefficient of *x_i_*, *β_0_* is the constant, and *ɛ* is the residual.

In the two logistical models, independent variables were the four social characteristic indicators in LSOAs. For each independent variable, the most affluent group served as the reference group. For example, when the employment variable was evaluated, all LSOAs in the most affluent group served as the reference category. One concern may be present on the unbalanced group sizes in the tweets. A rule of thumb for logistic models suggests a minimum of ten outcome events per predictor variable (EPV) [46,47]. Other studies [48,49] argued that this one-in-ten rule may be conservative. In particular, Vittinghoff and McCulloch [49] reported that 92.8% of scenarios with 5–9 EPV are within acceptable levels through a simulation study. In our case, the March and June incidents present EPVs of 25 and 7, respectively. Therefore, interpreting the June model may take caution. We used the Hosmer and Lemeshow test to examine the goodness of fit of the logistic regression models. The test returns a Hosmer–Lemeshow Chi-squared and its *p*-Value. The *p*-Value is derived by comparing the computed Hosmer–Lemeshow statistic to a Chi-squared distribution. A *p*-value less than 5% suggests that a model is a poor fit [50]. In addition, because of the imbalanced proportion between negative tweets and other tweets, we used the receiver operating characteristic (ROC) curve [51,52] to examine the discrimination ability of the two logistic regression models. The ROC curve plots the sensitivity (true positive) against specificity (true negative) for all possible cutoff points. In this research, a false positive occurs when a tweet is classified as presenting negative emotion but does not actually have negative emotional content. The area under the ROC curve ranges from 0.5 to 1 with larger values suggesting a better fit. The Nagelkerke pseudo-R Square was used to examine the magnitude of the model to explain the outcomes, that is, the percentage of the negative tweets estimated by the model [50,53]. All statistical analyses were carried out using IBM SPSS Statistics version 25 (IBM Corp, Armonk, NY, USA).

## 3. Results

**Figure 1:**
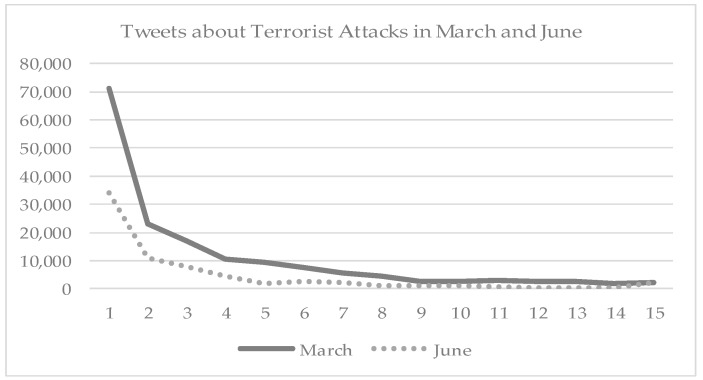
Number of Tweets on Terrorist in March and June 2017.

A total number of 237,035 tweets, regardless of being geotagged, were collected in two weeks following the two attacks, including 165,881 in March and 71,154 in June, respectively. The number of tweets grew rapidly at the beginning of the incidents, usually reaching the highest number on the first few days of an incident, and later gradually decreasing with time (Figure 1).

In general, new tweets became rare after two weeks and most were retweets of previous messages. However, responding to the second attack, tweets dropped quickly in one week and became very rare after. Among the geotagged tweets, there were 2183 geotagged tweets of which a total of 1,056 were in the United Kingdom, including 717 in March and 339 in June (Table 2). The majority of the tweets cast the news. A small number showed negative emotions, 100 (13.95%) in March and 28 (8.26%) in June, respectively. Compared to the March incident, the attack in June received less attention among tweet users, and the negative emotions became less prevalent as well. 

**Figure 2:**
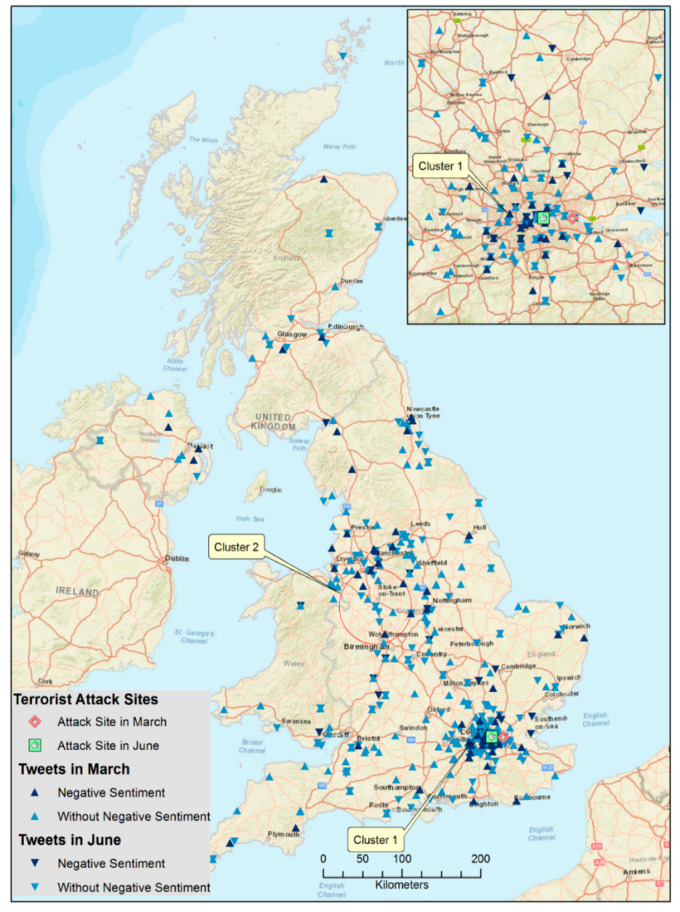
Clustering of Geotagged Tweets in the United Kingdom.

Mapping the tweet locations (Figure 2) reveals that most of the tweets were sent from England, and most of them were clustered in the southeast area, which is in centered around the City of London where the two attacks occurred. The rest were scattered in Northern Ireland, Scotland, and Wales. The space-time permutation model identified two statistically significant clusters of negative tweets after the March attack. The first was in London (Figure 2), taking place on 1 April 2017 from 19:00 to 20:00, ten days after the attack. There were 31 negative tweets in the same location. 

A close examination of the tweets suggested that these present the same message from the same twitter account, even though the timestamps associated with these tweets were different in the one-hour period. The motivation behind the user’s repeated tweeting of the same message was unclear. Therefore, interpreting this cluster takes caution. The second cluster of negative tweets was a circular area with a radius of 45 kilometers between Manchester and Birmingham, approximately 200 km away from London. Tweets in this cluster were sent between 25 March 2017 at 15:00 and 26 March 2017 at 1:00, three days after the attack. It included six negative tweets among 27 tweets resulting in 22.2% presenting negative emotions. When the other two search window sizes were alternated, SatScan consistently reported these two significant clusters. When the same model was applied to the tweets in June, no significant clusters were detected.

The summary statistics (Table 3) described the interaction of tweeting activities and social characteristics of communities in the United Kingdom in the two incidents separately.

Regarding the March incident, more deprived communities from economic perspectives, represented by the 1st and 2nd quartiles of the income and employment categories, generated fewer tweets. In particular, the most deprived communities generated approximately half the tweets of what the most affluent communities sent. Tweets from communities with better educational attainment were more than twice than the counterparts in the education domain. From the crime risk perspective, i.e., the risk of personal or material victimization, moderately deprived communities led the number of tweets. In comparison to the March incident, geotagged tweets related to the June incident declined quickly from 717 to 339. In general, tweets from the deprived groups dropped more than these from the affluent communities. This decrease was present in all four domains of social characteristics, as evidenced in the percentage of decrease (Table 3).

The regression result (Table 4) sheds light on the likelihood of presenting negative emotions in tweets associated with social characteristics. In tweets related to the March incident, negative emotions were more prevalent in the deprived communities, as can be seen in the rates of negative emotions across the income, employment, and education domains. Particularly in the education domain, the likelihood of tweeting negatively in moderately deprived communities significantly increased compared to the most affluent cohorts (odds ratio of 2.87 and *p* < 0.05). Pertaining to the tweets after the June incident, the rates of negative emotions across the four domains declined in general and the rate difference within a domain diminished. The regression model on the June incident, in fact, suggested that deprived communities in light of the crime risk had significantly decreased the likelihood of presenting negative tweets compared to the most affluent cohorts. However, interpreting the effect of crime risk shall be cautious because of the small number of negative tweets (*n* = 28) related to the June incident. 

**Figure 3:**
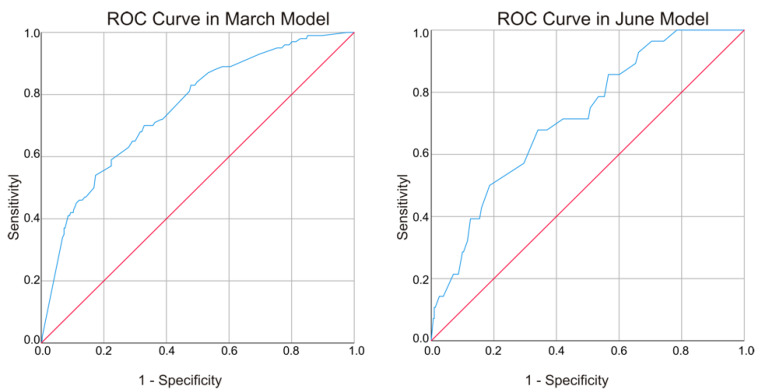
ROC curves in the two models. Note diagonal segments are produced by ties.

The model performance was evaluated in three aspects. The Hosmer and Lemeshow test in the two models returned *p*-values of 0.946 and 0.358, respectively. The corresponding Chi-square values were 2.799 and 8.814, suggesting that the number of negative tweets was not significantly different from those predicted by the models and that the overall fit of the two models was good. The area under the ROC curve was 0.754 with a 95% confidence interval (0.703–0.804) in the March model and 0.718 with a 95% confidence interval (0.626–0.810) in the June model (Figure 3). Both models suggested fair tests of separating the tweets being tested into those with and without negative emotions. In addition, the March model returned slightly better accuracy than the June model. In both curves, the area under the curve was significantly different from 0.5 since the *p*-value was less than 0.05, suggesting that the two models classified the tweets significantly better than by chance. The Nagelkerke R Square values of 0.186 in Model 1 and 0.11 in Model 2 suggested that the models explained roughly 18.6% and 11% of the variation in the negative tweets in March and June, respectively.

## 4. Discussion

Violent events result in substantial adverse consequences in mental health [3,4,5]. As geopolitical tensions escalated intimately interwoven with domestic and regional conflict, terrorism, and dysfunction of political powers, violence-introduced trauma may continue to have a devastated impact on the public [22]. This study contributes to this area of research by proposing a framework to rapidly detect geographically concentrated emotional reactions after the two consecutive terrorist attacks using social media data in the GIS. We postulated that various social characteristics of communities would be influential in explaining the elevated likelihood of such negative mental reactions in the aftermath of terrorist attacks. 

To our knowledge, this is the first study to document the mental reactions in a sample exposed to consecutive terrorist attacks in London in 2017. As such, we are unable to compare the prevalence documented here to other studies. Our research revealed elevated attentions of the violence and detected clusters of negative emotions, such as fear, sadness, and anger in the aftermath of these disasters. Specifically, the space-time model suggested two clusters in March, one was around the attack sites on 1 April 2017, ten days after the attack, and the other was a region between Manchester and Birmingham, three days after the incident. This is in line with a previous study [3] identifying elevated fear and sadness after the terrorist attacks in Paris in 2015. Both studies evidence the emergence of concentrated emotional responses in the aftermath of a man-made disaster. The findings agree with previous research [4,54], suggesting a significant increase in fear, distress, and severe stress symptoms associated with the terrorist attack sites. 

Furthermore, our research suggested that the terrorist attacks had a profound impact on the society, as evidenced by the second cluster, far beyond the city where the attacks occurred. It supports the previous studies [55,56] that although terrorism brings physical injuries to only a small part of society, its aftermath can reach far and broad with a period of hardship and stress. It is expected that people in the immediate area directly hit by an attack are prone to both physical and mental suffering, on which many research and policies have applaudingly focused on. In fact, areas far away from the attack sites may be perilous with negatively impacted victims and hitherto deserve effort to examine. On 19 June 2017, 16 days after the June London Bridge attack, Darren Osborne of Cardiff, the capital of Wales approximately 250 km from London, drove a van into pedestrians near Finsbury Park Mosque. This Finsbury Park attack, believed to be a revenge attack against Muslims responding to recent Islamist attacks [57,58], highlights the need for understanding the geographic distribution of negative emotions. Despite that the cluster sizes were relatively small compared to all extracted geotagged tweets, using refined social media data, pinning the specific locations may identify where early negative emotional reactions are present. As suggested by previous research [3,7,59,60], these early responses may be predictive of long-term mental health needs and formulate affected areas that require additional surveillance and study.

Another novelty of this study lies in its insights in temporal changes in mental reactions responding to reoccurring violence in a short time period. A plethora of studies examined PTSD longitudinally [9,10,61]. Our research is uniquely positioned, given that the two terrorist attacks were similar in nature and took place in the same city within only two months. The responses from tweet users, therefore, were more comparable in contrast to disasters in different countries or in the same country but in different years. Our findings suggest that attention to reoccurring disasters, even when it is the same kind and in the same city, decayed faster than the very first incident. Since terrorist organizations use terror as a “marketing” tool to tactically gain political or social objectives, the spike of tweets at the beginning of the two attacks may be interpreted as a measure of the terrorism “success” [17,62]. The two abruptly and horrific attacks, like other terrorists’ violent acts, made everyone feel at risk [63]. However, the number of tweets faded out consistently in two weeks, suggesting that the terrorists failed to provoke a prolonged reaction in the targeted audience. In fact, the reoccurring attack in June was far less “effective” than the first. The reasons are certainly multifactorial, but resilience [5,55], low-risk perception of less direct exposure [10], and coping and social support [64,65] could contribute to the generally placid reaction in social media. 

Besides the decrease in reactions, this research provides insights into the social characteristics of the communities where the messages were tweeted. In light of the digital divide highlighted in previous research [66,67], our findings reinforced the digital disadvantage among Twitter users in communities being poorer, less employed, and less educated. Despite the uneven tweeting, the negative emotions were more pervasive in the deprived cohorts, emphasizing the “double disadvantage” of living in the underprivileged neighborhoods. This socioeconomic disadvantage calls on the further investigation with individual measures of tweet users after accounting for confounders.

The implications of our research are twofold. On the one hand, geotagged social media data, if applied in real-time, could be used to rapidly detect emerging areas of emotional reactions over space and time in the aftermath of disasters [3,16]. The use of social media data is a cost-effective way over traditional surveys to understand how negative emotions occur in a group of people in a specific area over a limited time period after a disaster. If these clusters are of sufficient size, they may warrant further investigation and resources for follow-up. On the other hand, policies in the use of social media data need to take into consideration the digital disadvantage of the underprivileged. As social media data gains its popularity, policy implementations based on their usage would need to pay more attention to the issue of the under-representativeness of these users. 

Several limitations should be considered when interpreting the findings of this study. First, this study was based on only a small sample of all the tweets. Only a small portion of the tweets carry a geotag, which is a common challenge acknowledged in mapping social media data [68,69]. The small sample size restricted our ability to extract a specific type of emotions, such as anger, over others to provide more detailed information and may affect the regression analysis. The imbalanced proportion between negative tweets and other tweets limited the discrimination ability of the logistic regression models. Second, the tweets were further limited by the keywords. Our attempts to retrieve tweets without keywords were prohibited by the rate limit set by Twitter and our computer limitation. The distribution of emotions may change if all tweets would have been captured and geotagged. Third, the manual classification of each tweet’s content is subjective and may introduce our researchers’ bias. Fourth, this study is limited in using English tweets. Further research is warranted to use other types of social media data (e.g., Facebook) or non-English languages. Last but not least, interpreting the findings shall keep awareness of the representative issue due to the differences in demographics of Twitter users [66,70].

## 5. Conclusions

Terrorism poses a significant physical and psychological health threat to the public. Understanding the pronounced emotional reactions in real-time after a disaster provides proactive guidance for the provision of counseling services for mental health [3,71]. This study analyzed the geotagged Twitter data in the United Kingdom to identify the clusters of negative emotional reactions in space and time. It also examined the social characteristics of communities associated with negative tweets. This research was conducted using a single social media platform in one language. While the findings provide insights into concentrated mental distress, further research may benefit from developing solutions to explore various social media types in multiple languages. Studies [3,31] including ours dichotomized social media data to consider various reactions (fear, sadness, anger, shock, confusion, disgust, shame, among others) as negative emotions. Large volumes of social media data coupled with more advanced data mining techniques are needed to discern the emotion types. Further classification of the data into multiple groups will give a finer-grained sense of certain kinds of negative emotions, providing more specific information to policymakers and health professionals to respond accordingly. As disasters continue in various forms, such as the ongoing COVID-19 pandemic, efficient mining of social media data coupled with comprehensive surveillance, screening, and care strategies would protect the mental well-being of the public in the event of consecutive and prolonged disasters.

## Figures and Tables

**Table 1:** Sample tweets.

Longitude/Latitude	Date	Time	Tweet	Negative
(−0.775435, 51.279904)	3/23/2017	20:58:23	So sad watching the vigil in London today. Our thoughts and amp; prayers are with everyone who was affected by yesterday’s attack	1
(−3.0812071, 51.549936)	3/27/2017	17:32:17	I think they are sensible and amp; very necessary to stop a tory government exploiting Brexit to attack people’s rights…	1
(−0.297251, 51,685439)	3/29/2017	6:56:29	the last legs’ response to the London attack is f—brilliant ????	1
(−0.15191, 51.410792)	6/6/2017	22:05:35	r.i.p ???? I had tears in my eyes walking past where the attack was London you are beautiful ?? London strong	1
(−0.422572, 53.719616)	3/23/2017	21:32:50	Thank you to all my twitter friends for your support after the attack on London	0
(−0.213503, 51.512805)	3/30/2017	17:44:10	London attack: Khalid Masood ‘died from shot to chest’	0
(−0.187894, 51.483718)	6/6/2017	9:10:17	A minute’s silence will be held at 11 am today in remembrance of those who died in the London Bridge attack??	0

**Table 2:** Number of geotagged tweets in the UK.

Title	Geotagged Tweets (Number)	Negative Tweets (Number)	Percent of Negative Tweets
March	717	100	13.95%
June	339	28	8.26%

**Table 3:** Descriptive statistics of the social characteristics of the LSOAs in tweeting activities.

Social Characteristics	March		June		Decrease ^a^
	# Tweets	% Tweets	# Tweets	% Tweets	
Income					
Most deprived	117	16.3	48	14.2	59.0%
Moderate deprivation	158	22.0	71	20.9	55.1%
Moderate affluence	240	33.5	110	32.5	54.2%
Most affluence	202	28.2	110	32.4	45.5%
Employment					
Most deprived	107	14.9	41	12.1	61.7%
Moderate deprivation	112	15.6	60	17.7	46.4%
Moderate affluence	269	37.5	108	31.9	59.9%
Most affluence	229	32.0	130	38.3	43.2%
Education					
Most deprived	43	6.0	31	9.2	27.9%
Moderate deprivation	153	21.3	58	17.1	62.1%
Moderate affluence	277	38.6	133	39.2	52.0%
Most affluence	244	34.1	117	34.5	52.0%
Crime					
Most deprived	161	22.4	90	26.5	44.1%
Moderate deprivation	251	35.0	100	29.5	60.2%
Moderate affluence	159	22.2	79	23.3	50.3%
Most affluence	146	20.4	70	20.7	52.1%

^a^ Values in the decrease column were calculated using the difference in the number of tweets in a quartile divided by the number of tweets for the March incident from the same quartile. # Tweets is the number of tweets in a category.

**Table 4:** The risk of negative emotions in relation to social characteristics.

Social Characteristics	March Incident		June Incident	
	Negative Rate ^a^	Odds Ratio (95% CI ^b^)	Negative Rate ^a^	Odds Ratio (95% CI ^b^)
Income				
Most deprived	36	2.4 (0.39, 14.99)	6	12.23 (0.43, 345.15)
Moderate deprivation	6	0.32 (0.08, 1.26)	6	1.3 (0.15, 11.28)
Moderate affluence	10	0.48 (0.18, 1.25)	10	1.12 (0.29, 4.29)
Most affluence ^c^	12	1	9	1
Employment				
Most deprived	36	1.5 (0.22, 10.19)	5	0.03 (0.001, 1.49)
Moderate deprivation	13	2.05 (0.56, 7.53)	7	0.32 (0.04, 2.64)
Moderate affluence	9	1.95 (0.76, 4.98)	9	1.52 (0.43, 5.41)
Most affluence ^c^	10	1	9	1
Education				
Most deprived	9	0.49 (0.12, 2.06)	10	8.23 (0.74, 92.11)
Moderate deprivation	31	2.87 * (1.15, 7.16)	7	1.59 (0.34, 7.48)
Moderate affluence	10	1.1 (0.54, 2.24)	9	1.37 (0.48, 3.89)
Most affluence ^c^	9	1	8	1
Crime				
Most deprived	6	0.43 (0.15, 1.19)	3	0.12 * (0.02, 0.71)
Moderate deprivation	19	0.69 (0.31, 1.5)	5	0.23 * (0.06, 0.9)
Moderate affluence	16	1.44 (0.71, 2.89)	14	0.87 (0.31, 2.47)
Most affluence ^c^	12	1	13	1

^a^ Number of negative tweets per 100 tweets in a category. ^b^ CI, confidence interval. ^c^ Reference category in the model serves as the basis for comparison. * *α* = 0.05.

## References

[1] Medina R.M., Siebeneck L.K., Hepner G.F. (2011). A Geographic Information Systems (GIS) Analysis of Spatiotemporal Patterns of Terrorist Incidents in Iraq 2004–2009. Stud. Confl. Terror..

[2] Guo W.S. (2019). Common statistical patterns in urban terrorism. R. Soc. Open Sci..

[3] Gruebner O., Sykora M., Lowe S.R., Shankardass K., Trinquart L., Jackson T., Subramanian S.V., Galea S. (2016). Mental health surveillance after the terrorist attacks in Paris. Lancet.

[4] Dale M.T.G., Nissen A., Berthelsen M., Heir T. (2020). Post-traumatic stress reactions and doctor-certified sick leave after a workplace terrorist attack: Norwegian cohort study. BMJ Open.

[5] Lewis C.W. (2002). The terror that failed: Public opinion in the aftermath of the Bombing in Oklahoma City. Public Adm. Rev..

[6] Schuster M.A., Stein B.D., Jaycox L.H., Collins R.L., Marshall G.N., Elliott M.N., Zhou A.J., Kanouse D.E., Morrison J.L., Berry S.H. (2001). A national survey of stress reactions after the September 11, 2001, terrorist attacks. N. Engl. J. Med..

[7] Galea S., Nandi A., Vlahov D. (2005). The epidemiology of post-traumatic stress disorder after disasters. Epidemiol. Rev..

[8] North C.S., Nixon S.J., Shariat S., Mallonee S., McMillen J.C., Spitznagel E.L., Smith E.M. (1999). Psychiatric disorders among survivors of the Oklahoma City bombing. J. Am. Med. Assoc..

[9] Neria Y., Nandi A., Galea S. (2008). Post-traumatic stress disorder following disasters: A systematic review. Psychol. Med..

[10] Galea S., Ahern J., Resnick H., Kilpatrick D., Bucuvalas M., Gold J., Vlahov D. (2002). Psychological sequelae of the September 11 terrorist attacks in New York City. N. Engl. J. Med..

[11] Cvetojevic S., Hochmair H.H. (2018). Analyzing the spread of tweets in response to Paris attacks. Comput. Environ. Urban. Syst..

[12] Lin Y.R., Margolin D., Wen X.D. (2017). Tracking and Analyzing Individual Distress Following Terrorist Attacks Using Social Media Streams. Risk Anal..

[13] Yates D., Paquette S. (2011). Emergency knowledge management and social media technologies: A case study of the 2010 Haitian earthquake. Int. J. Inf. Manag..

[14] Muralidharan S., Rasmussen L., Patterson D., Shin J.H. (2011). Hope for Haiti: An analysis of Facebook and Twitter usage during the earthquake relief efforts. Public Relat. Rev..

[15] Gruebner O., Lowe S.R., Sykora M., Shankardass K., Subramanian S.V., Galea S. (2018). Spatio-Temporal Distribution of Negative Emotions in New York City After a Natural Disaster as Seen in Social Media. Int. J. Environ. Res. Public Health.

[16] Chen S.J., Mao J., Li G., Ma C., Cao Y.J. (2020). Uncovering sentiment and retweet patterns of disaster-related tweets from a spatiotemporal perspective—A case study of Hurricane Harvey. Telemat. Inform..

[17] Sandler T. (2003). Collective action and transnational terrorism. World Econ..

[18] Sandler T. (2011). New frontiers of terrorism research: An introduction. J. Peace Res..

[19] Crenshaw M. (2007). Explaining suicide terrorism: A review essay. Secur. Stud..

[20] National Academy of Science (2003). Preparing for the Psychological Consequences of Terrorism: A Public Health Strategy.

[21] National Consortium for the Study of Terrorism and Responses to Terrorism University of Maryland Global Terrorism Database. https://www.start.umd.edu/gtd/.

[22] Goldman O. (2011). The Globalization of Terror Attacks. Terror. Political Violence.

[23] Nugent J.L., Khashan A.S., Baker P.N. (2011). Reduced infant birth weight in the North West of England consequent upon ‘maternal exposure’ to 7/7 terrorist attacks on central London. J. Obstet. Gynaecol..

[24] Stecklov G., Goldstein J.R. (2004). Terror attacks influence driving behavior in Israel. Proc. Natl. Acad. Sci. USA.

[25] Norris F.H., Friedman M.J., Watson P.J., Byrne C.M., Diaz E., Kaniasty K. (2002). 60,000 disaster victims speak: Part, I. An empirical review of the empirical literature, 1981–2001. Psychiatry Interpers. Biol. Process..

[26] Baum A. (1990). Stress, intrusive imagery, and chronic distress. Health Psychol..

[27] Green B.L., Lindy J.D., Grace M.C., Gleser G.C., Leonard A.C., Korol M., Winget C. (1990). Buffalo Creek survivors in the second decade: Stability of stress symptoms. Am. J. Orthopsychiatry.

[28] Solomon S.D., Green B.L. (1992). Mental health effects of natural and human-made disasters. PTSD Res. Q..

[29] Coppersmith G., Dredze M., Harman C. Quantifying Mental Health Signals in Twitter. Proceedings of the Workshop on Computational Linguistics and Clinical Psychology: From Linguistic Signal to Clinical Reality.

[30] Twitter, Inc Rate Limiting. https://developer.twitter.com/en/docs/basics/rate-limiting.

[31] Sykora M., Jackson T., O’Brien A. (2013). Emotive ontology: Extracting fine-grained emotions from terse, informal messages. IADIS Int. J. Comput. Sci. Inf. Syst..

[32] Kaur H.J., Kumar R. Sentiment Analysis from Social Media in Crisis Situations. Proceedings of the 2015 International Conference on Computing, Communication & Automation (ICCCA).

[33] Khan F.H., Qamar U., Bashir S. (2016). eSAP: A decision support framework for enhanced sentiment analysis and polarity classification. Inf. Sci..

[34] Khan F.H., Qamar U., Bashir S. (2016). Senti-CS: Building a lexical resource for sentiment analysis using subjective feature selection and normalized Chi-Square-based feature weight generation. Expert Syst..

[35] Ji R.R., Cao D.L., Zhou Y.Y., Chen F.H. (2016). Survey of visual sentiment prediction for social media analysis. Front. Comput. Sci..

[36] Paltoglou G. (2016). Sentiment-Based Event Detection in Twitter. J. Assoc. Inf. Sci. Technol..

[37] Schumaker R.P., Jarmoszko A.T., Labedz C.S. (2016). Predicting wins and spread in the Premier League using a sentiment analysis of twitter. Decis. Support. Syst..

[38] Kulldorff M., Heffernan R., Hartman J., Assuncao R.M., Mostashari F. (2005). A space-time permutation scan statistic for the early detection of disease outbreaks. PLoS Med..

[39] Hochmair H., Cvetojevic S. (2014). Assessing the Usability of Georeferenced Tweets for the Extraction of Travel Patterns: A Case Study for Austria and Florida.

[40] Kulldorff M. (1999). An isotonic spatial scan statistic for geographical disease surveillance. J. Natl. Inst. Public Health.

[41] University of Essex, University of Manchester and Jisc Deprivation Data. https://census.ukdataservice.ac.uk/get-data/related/deprivation.aspx.

[42] U.K. National Health Service NHS: Business Definitions. https://www.datadictionary.nhs.uk/data_dictionary/nhs_business_definitions/l/lower_layer_super_output_area_de.asp?shownav=1.

[43] U.S. Census Bureau United States Census Bureau Glossary on Census Divisions and Census Regions. https://www.census.gov/programs-surveys/geography/about/glossary.html.

[44] Budenz A., Klassen A., Purtle J., Tov E.Y., Yudell M., Massey P. (2019). Mental illness and bipolar disorder on Twitter: Implications for stigma and social support. J. Ment. Health..

[45] Dutta H., Kwon K.H., Rao H.R. (2018). A system for intergroup prejudice detection: The case of microblogging under terrorist attacks. Decis. Support. Syst..

[46] Harrell F.E.J., Lee K.L., Mark D.B. (1996). Multivariable prognostic models: Issues in developing models, evaluating assumptions and adequacy, and measuring and reducing errors. Stat. Med..

[47] Peduzzi P., Concato J., Kemper E., Holford T.R., Feinstein A.R. (1996). A simulation study of the number of events per variable in logistic regression analysis. J. Clin. Epidemiol..

[48] Steyerberg E.W., Eijkemans M.J.C., Harrell F.E.J., Habbema J.D.F. (2000). Prognostic modelling with logistic regression analysis: A comparison of selection and estimation methods in small data sets. Stat. Med..

[49] Vittinghoff E., McCulloch C.E. (2007). Relaxing the rule of ten events per variable in logistic and cox regression. Am. J. Epidemiol..

[50] Hosmer D.W., Lemeshow S.A., Sturdviant R.X. (2013). Applied Logistic Regression.

[51] Powers D.M.W. (2011). Evaluation: From precision, recall and F-measure to ROC, informedness, markedness & correlation. J. Mach. Learn. Technol..

[52] Fawcett T. (2006). An introduction to ROC analysis. Pattern Recognit. Lett..

[53] Long J.S. (1997). Regression Models for Categorical and Limited Dependent Variables.

[54] Mahat-Shamir M., Hoffman Y., Pitcho-Prelorentzos S., Hamama-Raz Y., Lavenda O., Ring L., Halevi U., Ellenberg E., Ostfeld I., Ben-Ezra M. (2018). Truck attack: Fear of ISIS and reminder of truck attacks in Europe as associated with psychological distress and PTSD symptoms. Psychiatry Res..

[55] Wrzesniewski A. (2002). “It’s not just a job”: Shifting meanings of work in the wake of 9/11. J. Manag. Inq..

[56] Inness M., Barling J., Barling J., Kelloway E.K., Frone M.R. (2005). Terrorism. Handbook of Work Stress.

[57] Rawlingson K. (2018). Darren Osborne Jailed for Life for Finsbury Park Terrorist Attack. https://www.theguardian.com/uk-news/2018/feb/02/finsbury-park-attack-darren-osborne-jailed.

[58] Mortimer C. (2017). Darren Osborne: Family of Man Arrested after Finsbury Park Mosque Terror Attack Says He Is ‘Troubled’ but ‘not Racist’. https://www.independent.co.uk/news/uk/crime/finsbury-park-mosque-terror-attack-muslims-darren-osborne-van-driver-family-neighbour-troubled-a7798256.html.

[59] Goldmann E., Galea S. (2014). Mental Health Consequences of Disasters. Ann. Rev. Public Health.

[60] Bryant R.A. (2011). Acute stress disorder as a predictor of posttraumatic stress disorder: A systematic review. J. Clin. Psychiatry.

[61] Curtis A., Mills J.W., Leitner M. (2007). Katrina and vulnerability: The geography of stress. J. Health Care Poor Underserved.

[62] Crenshaw M. (2014). Terrorism Research: The Record. Int. Interact..

[63] Sandler T. (2015). Terrorism and counterterrorism: An overview. Oxf. Econ. Pap. New Ser..

[64] Zara A. (2020). Grief intensity, coping and psychological health among family members and friends following a terrorist attack. Death Stud..

[65] Weinberg M. (2018). The Mediating Role of Posttraumatic Stress Disorder with Tendency to Forgive, Social Support, and Psychosocial Functioning of Terror Survivors. Health Soc. Work.

[66] Blank G., Lutz C. (2017). Representativeness of Social Media in Great Britain: Investigating Facebook, LinkedIn, Twitter, Pinterest, Google plus, and Instagram. Am. Behav. Sci..

[67] Xiao Y., Huang Q.Y., Wu K. (2015). Understanding social media data for disaster management. Nat. Hazards.

[68] Tsou M.H. (2015). Research challenges and opportunities in mapping social media and Big Data. Cartogr. Geogr. Inf. Sci..

[69] Han S.Y., Tsou M.H., Clarke K.C. (2015). Do global cities enable global views? Using Twitter to quantify the level of geographical awareness of U.S. Cities. PLoS ONE.

[70] Sloan L., Morgan J. (2015). Who Tweets with Their Location? Understanding the Relationship between Demographic Characteristics and the Use of Geoservices and Geotagging on Twitter. PLoS ONE.

[71] Whalley M.G., Brewin C.R. (2007). Mental health following terrorist attacks. Br. J. Psychiatry.

